# Publication Non Grata: The Challenge of Publishing Non-COVID-19 Research in the COVID Era

**DOI:** 10.7759/cureus.11403

**Published:** 2020-11-09

**Authors:** Judy Shan, Dustin Ballard, David R Vinson

**Affiliations:** 1 Division of Research, Kaiser Permanente, Oakland, USA; 2 Emergency Medicine, Kaiser Permanente San Rafael Medical Center, San Rafael, USA; 3 Emergency Medicine, Kaiser Permanente Sacramento Medical Center, Sacramento, USA

**Keywords:** covid 19, publication trends, publication bias, pandemics, research trends, corona virus disease 2019, research and publishing, publication ethics

## Abstract

Objectives: We aimed to determine publication trends in leading clinical research journals (impact factor >20) during the rise of the coronavirus disease 2019 (COVID-19) pandemic and to check for an increase in publication times of non-COVID-19 original research articles.

Methods: We collected publication data from five print-based medical journals and one online journal--JAMA: The Journal of the American Medical Association, The Lancet (Lancet), The New England Journal of Medicine, Annals of Internal Medicine, The BMJ (BMJ), and BMC Medicine (BMC Med)--for the December 2019 through May 2020 period. We categorized each article as either "COVID-19-related" or "non-COVID-19-related". When available, we further extracted data on submission-to-acceptance dates and acceptance-to-publication dates for original research articles for the January through July 2019 and January through July 2020 periods. We compared the time from submission to publication for non-COVID-19 original research articles during the two periods and tested for statistical significance with a one-tailed Wilcoxon rank-sum test.

Results: We found that non-COVID-19-related articles began decreasing in volume as COVID-19-related articles increased. In BMJ and Lancet, the COVID-19-related articles began overtaking the non-COVID-19-related articles in number during April and May 2020. However, COVID-19-related primary research articles only began consistently appearing in journal issues during May 2020. Only BMJ and BMC Med publicly recorded complete data regarding their publication timelines. After removing outliers, we found that the mean time from submission to publication for articles published in BMJ from January through July 2019 was 204 days (median: 194 days; IQR: 163-236), and from January through July 2020 was 223 days (median: 218 days; IQR: 177-261) (p=0.04). In BMC Med, the mean time from submission to publication from February through July 2019 was 153 days (median: 150 days; IQR: 123-181), and from February through July 2020 was 163 days (median: 157 days; IQR: 132-191) (p=0.06).

Conclusion: We discovered a steadily increasing trend in the percentage of COVID-19-related articles and a concomitant decreasing trend in the percentage of non-COVID-19-related articles published in high-impact print journals during the period from December 2019 through May 2020. For non-COVID-19-related articles published in BMJ, we found a statistically significant increase upon comparing the submission-to-publication times for the period from January through July 2020 with the submission-to-publication times for the period from January through July 2019.

## Introduction

Time-lag bias and publication bias can lead to barriers in research transparency and integrity [1]. Previous studies have shown that publication bias can result from a low interest in study results among editors, conflicts of interest that suppress publication of data that do not align with certain agendas, non-significant study results, and other competing commitments [2-5].

Throughout the current coronavirus disease 2019 (COVID-19) pandemic expedited review and publication of COVID-19 research and opinion articles have been essential to the dissemination of crucial information. The rapid course of the pandemic and urgency from the public have pressured journals to publish as quickly and widely as possible. However, there have been issues associated with hastened article review timelines and the subsequent publication of unverified information [6,7]. This misinformation can result in mismanagement of the disease process and misguided development of public health guidelines [6].

Despite the necessity of communicating information regarding the current pandemic to public health officials and the rest of the community, it is also important to not overlook research that is non-COVID-19-related. The onset of the pandemic does not diminish the need for the distribution of high-quality research related to other disease processes and conditions. Our study evaluates displacement in the publication of non-COVID-19-related research articles, both in terms of quantity and submission-to-publication times. We aim to determine publication trends in leading clinical research journals during the rise of the COVID-19 pandemic and to explore whether there is an increase in time from submission to publication for non-COVID-19 original research articles compared to the same period of the prior year.

## Materials and methods

We collected publication data from five major print medical journals (impact factor>20)--JAMA: The Journal of the American Medical Association (JAMA), The Lancet (Lancet), The New England Journal of Medicine (N Engl J Med), Annals of Internal Medicine (Ann Intern Med), and The BMJ (BMJ)--for the December 2019 through May 2020 period. We extracted data on the number of articles published per issue in each of the following categories: "Original research", "Reviews", "Editorials", "Comments", "Correspondence/Letters", "Case Reports", and "Other". We categorized each article as either "COVID-19-related" or "non-COVID-19-related".

We also extracted data on submission-to-publication times for non-COVID-19 original research articles, when reported. The purpose of this was to compare the submission-to-publication times for the January through July 2020 period with those for the January through July 2019 period. We further extracted similar data on original research articles published in the BMC Medicine (BMC Med), an online-only medical journal, for the February through July 2019 and February through July 2020 periods. We plotted the data using boxplots and excluded outliers. We then compared the submission-to-publication times for the specified periods within each journal. We tested for statistical significance using a one-tailed Wilcoxon rank-sum test. A p-value of <0.05 was accepted as statistically significant.

## Results

**Figure 1:**
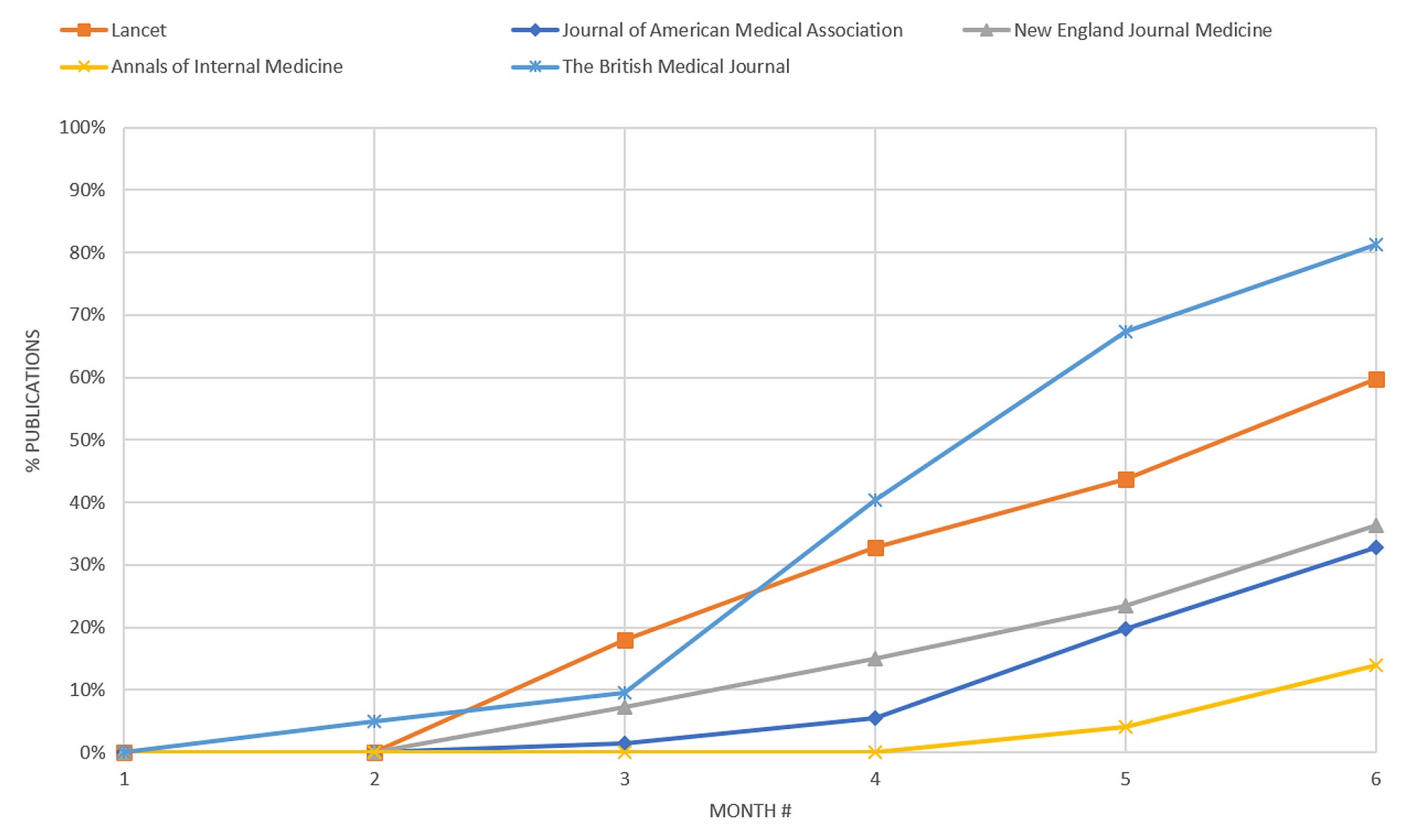
Percentage of COVID-related publications appearing in five select journals from December 2019 through May 2020

We found that COVID-19-related articles started appearing in late January/early February 2020. COVID-19-related articles began overtaking non-COVID-19-related articles in Lancet and BMJ in April and May 2020, respectively. In The Lancet, the percentage of COVID-19-related articles grew from 0% in December 2019 to 18% in February 2020, then to 44% in April 2020, and then to 60% in May 2020. In BMJ, the percentage of COVID-19-related articles grew from 0% in December 2019 to 10% in February 2020, then to 67% in April 2020, and then to 81% in May 2020. The percentages of COVID-19-related articles in the remaining journals in May 2020 were as follows: JAMA (33%), N Engl J Med (36%), and Ann Intern Med (14%) (Figure 1).

However, COVID-19-related articles only began appearing consistently under the category “primary research” in journal issues during May 2020. A majority of the published COVID-19-related articles fell into various categories other than original research, such as “editorials”, “comments”, “correspondence/letters”, and “other”. The “other” category included news, short essays, and viewpoints/perspectives.

For the publication delay analysis, only BMJ among the five print-based journals reported complete publication timeline data. We excluded four outlier articles from 2019 (1%) and three from 2020 (6%). The mean time from submission to publication for non-COVID-19-related original research articles published in BMJ from January through July 2019 was 204 days (median: 194 days; IQR: 163-236), and from January through July 2020 was 223 days (median: 218 days; IQR: 177-261). The difference in the median submission-to-publication times between 2019 and 2020 was 24 days (p=0.04).

Unlike leading print journals, the majority of articles published in BMC Med were original research articles. The number of non-COVID-19 articles did not decrease with the onset of COVID-19. In fact, the total number of articles increased with the addition of COVID-19-related studies. For our publication delay analysis, we excluded one outlier article from 2019 (1%) and four from 2020 (4%). The mean time from submission to publication from February through July 2019 was 153 days (median: 150 days; IQR: 123-181), and from February through July 2020 was 163 days (median: 157 days; IQR: 132-191) (Table 1). We found a difference of seven days in the median submission- to- publication times between 2019 and 2020 (p=0.06).

**Table 1:** Table 1: Submission-to-publication data for non-COVID original research articles published in BMJ and BMC Med *Note: time periods studied were based on when COVID-related articles began appearing in each journal. BMJ: The BMJ; BMC Med: BMC Medicine.

		BMJ	BMC Med
Time periods studied*		January 2019-July 2019 and January 2020-July 2020	February 2019-July 2019 and February 2020-July 2020
Mean time from submission to publication (days)	2019	204 (median: 194)	153 (median: 150)
	2020	223 (median: 218)	163 (median: 157)
Number of non-COVID original research articles	2019	62	77
	2020	44	94

## Discussion

We discovered a steady, increasing trend in the number of COVID-19-related articles published and a general decreasing trend in the percentage of non-COVID-19-related articles across leading print journals for the December 2019 through May 2020 period. This raises questions about whether the decrease in non-COVID-19 articles was due to fewer non-COVID-19 article submissions, publication bias on the part of the journals, or a mixture of both. While we did find a significant increase in the submission-to-publication times of non-COVID-19 original research articles published in BMJ, when the period encompassing the rise of the COVID-19 pandemic was compared with the same period in 2019, it is unclear whether this increase should be attributed to slower author response or a lag in journal feedback/acknowledgment.

We also attempted to determine if a publication delay was present for a non-print journal similar to the delay discovered for print journals, especially since the staff in the former type of journals is not constrained to work from a fixed physical space. Our findings based on publication data available for BMC Med, an online-only medical journal, revealed a smaller increase in the median submission-to-publication time (between 2019 and 2020) than the increase observed for BMJ (7 vs 24 days). The different delays observed for BMJ and BMC Med could be attributed to several factors, such as availability of peer reviewers and journal publishing protocols and goals. However, our study was not able to examine these factors, though they are worth exploring.

**Figure 2:**
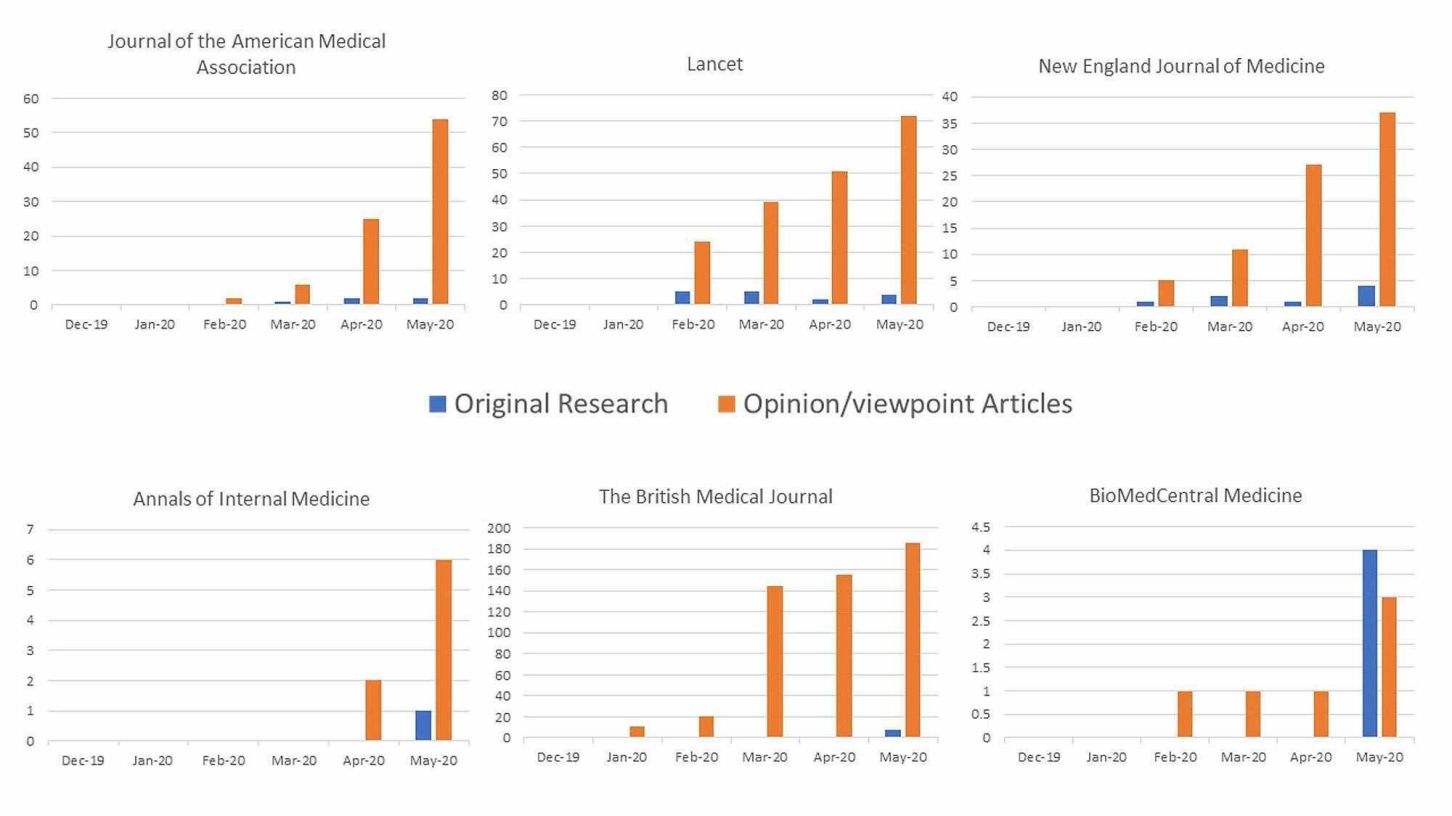
COVID-19 articles: original research versus opinion/viewpoint pieces

What we do know is that there has been a definite surge in the submission of COVID-19-related articles to journals for publication: the N Engl J Med has reported receiving up to 40 COVID-19-related articles in a single day, while JAMA has reported a daily total of up to 235 COVID-19-related article submissions [6]. Multiple leading publishers (Elsevier, Oxford University Press, Sage Publications, Wiley, etc.) have offered priority handling and expedited reviews for COVID-19-related articles [8]. Estimates from early-summer 2020 have shown the volume of COVID-19-related publications volume to be doubling roughly every 20 days [8]. However, not all of these publications are necessarily significant in broadening medical knowledge regarding COVID-19. A recent study by Elgendy et al. revealed that the number of “opinion” articles was greater than three times the number of primary research articles published from February through April 2020 [9]. They highlighted that although the current pandemic circumstances necessitate the dissemination of important insights to guide research and disease management efforts, there may be some redundancy in the publication content [9]. In a study characterizing COVID-19-related articles published during the first three months of the pandemic, Girolamo et al. brought up concerns that the overwhelming number of secondary articles (articles without original research findings) was diluting the original information on the pandemic [10]. We found a similar publication pattern in our study, where the ratio of opinion to original research articles remained quite high throughout the study inclusion period in all journals except for BMC Med (Figure 2). Another report found that a broad search for COVID-19-related articles in the COVID-19 Open Research Dataset (called CORD-19), revealed only 2% (2,600/140,000) of them to be clinical studies involving COVID-19 patients [8].

Due to the pandemic, the scientific community is currently generating a large number of COVID-19-related manuscripts, a significant proportion of which are opinion and viewpoint pieces. While there is undoubtedly a need for efficient dissemination of medical knowledge regarding COVID-19, it is crucial to recognize that not all articles related to COVID-19 are equal. The sheer volume of submitted manuscripts may make it more difficult for editors and peer reviewers to perform a proper review. It has already resulted in the publication of studies with unverified information and questionable findings [11-14]. Kurth et al. have suggested potential solutions to facilitate a more effective review process in times of limited publication resources with high demand [11]. These include utilizing a parallel reviewing system of “editorial triage” for both COVID-19-related and non-COVID-19-related submissions (such that non-COVID-19-related articles are less likely to be overlooked), sharing peer review comments and suggestions with authors as soon as they are in, so that authors may begin revisions sooner, and prioritizing the publication of papers with higher transparency and reproducibility [11].

Moreover, publishing excessive amounts of COVID-19-related articles may potentially hinder or delay the publication of high-quality research in other medically relevant fields and specialties. The urgency and proximity of the COVID-19 pandemic should not diminish the need for publication of research findings on other major infectious diseases, such as HIV/AIDS, malaria, and tuberculosis [15]. While there is currently an understandable research priority placed on COVID-19, it is also imperative to strike a balance in the delivery of relevant and valuable scientific evidence across a broader spectrum. We hope to bring awareness to this situation such that non-COVID-19-related research will not be overlooked amid the abundance of COVID-19-related articles.

This study is limited by the number of journals we included. The publication delay analysis was also limited because complete publication timeline details were available only for BMJ and BMC Med.

## Conclusions

The data on five leading clinical research print journals pointed to an increasing trend in the number of COVID-19 articles published over the six months from December 2019 through May 2020. This increase was accompanied by a corresponding decrease in non-COVID-19-related publications. We also found a significant increase in the submission-to-publication times of non-COVID-19-related articles published in BMJ when comparing the January through July 2020 period with the January through July 2019 period. Simultaneous with the publication delay of non-COVID-19-related articles, the publication of COVID-19-related articles was significantly expedited. Meanwhile, we found less delay in BMC Med, an online-only journal. Our report may help inform researchers, editors, and publishers about potential publication biases in certain journals during medical crises and inform them about potential avenues for the dissemination of their research.
